# Circulating lymphocytes and monocytes transcriptomic analysis of patients with type 2 diabetes mellitus, dyslipidemia and periodontitis

**DOI:** 10.1038/s41598-020-65042-9

**Published:** 2020-05-18

**Authors:** Sâmia C. T. Corbi, Jaira F. de Vasconcellos, Alliny S. Bastos, Diego Girotto Bussaneli, Bárbara Roque da Silva, Raquel Alves Santos, Catarina S. Takahashi, Cristiane de S. Rocha, Benilton de Sá Carvalho, Cláudia V. Maurer-Morelli, Silvana R. P. Orrico, Silvana P. Barros, Raquel M. Scarel-Caminaga

**Affiliations:** 1 Department of Diagnosis and Surgery, School of Dentistry at Araraquara, UNESP- São Paulo State University, Araraquara, 14801385 SP Brazil; 2 Department of Morphology, Genetics, Orthodontics and Pediatric Dentistry, School of Dentistry at Araraquara, UNESP- São Paulo State University, Araraquara, 14801385 SP Brazil; 3 Molecular Genomics and Therapeutics Section, Genetics of Development and Disease Branch, National Institute of Diabetes and Digestive and Kidney Diseases, National Institutes of Health, 10 Center Drive, Building 10, Room 9D11, Bethesda, MD 20892 USA; 4 Present Address: Department of Surgery, Uniformed Services University of the Health Sciences and Henry Jackson Foundation for the Advancement of Military Medicine, Bethesda, MD USA; 5 Postgraduate Program in Sciences of the University of Franca, Franca, 14404600 SP Brazil; 6 Department of Genetics, Faculty of Medicine of Ribeirão Preto, USP – University of São Paulo, Ribeirão Preto, 14049900 SP Brazil; 7 Department of Biology, Faculty of Philosophy Sciences and Letters of Ribeirão Preto, USP –University of São Paulo, Ribeirão Preto, 14049900 SP Brazil; 8 Department of Medical Genetics and Medicine Genomics, University of Campinas – UNICAMP, Campinas, 13083-887 SP Brazil; 9 Department of Statistics, Institute of Mathematics, Statistics and Scientific Computing, University of Campinas, 13083-859 São Paulo, Brazil; 10 Department of Periodontology, University of North Carolina at Chapel Hill – UNC, School of Dentistry, Chapel Hill, NC USA; 11 Advanced Research Center in Medicine, Union of the Colleges of the Great Lakes (UNILAGO), São José do Rio Preto, SP 15030-070 Brazil

**Keywords:** Gene expression, Dental diseases, Genetics research

## Abstract

Type 2 diabetes mellitus (T2DM), dyslipidemia and periodontitis are frequently associated pathologies; however, there are no studies showing the peripheral blood transcript profile of these combined diseases. Here we identified the differentially expressed genes (DEGs) of circulating lymphocytes and monocytes to reveal potential biomarkers that may be used as molecular targets for future diagnosis of each combination of these pathologies (compared to healthy patients) and give insights into the underlying molecular mechanisms of these diseases. Study participants (n = 150) were divided into groups: (H) systemically and periodontal healthy (control group); (P) with periodontitis, but systemically healthy; (DL-P) with dyslipidemia and periodontitis; (T2DMwell-DL-P) well-controlled type 2 diabetes mellitus with dyslipidemia and periodontitis; and (T2DMpoorly-DL-P) poorly-controlled type 2 diabetes mellitus with dyslipidemia and periodontitis. We preprocessed the microarray data using the Robust Multichip Average (RMA) strategy, followed by the RankProd method to identify candidates for DEGs. Furthermore, we performed functional enrichment analysis using Ingenuity Pathway Analysis and Gene Set Enrichment Analysis. DEGs were submitted to pairwise comparisons, and selected DEGs were validated by quantitative polymerase chain reaction. Validated DEGs verified from T2DMpoorly-DL-P *versus* H were: *TGFB1I1*, *VNN1*, *HLADRB4* and *CXCL8*; T2DMwell-DL-P *versus* H: *FN1*, *BPTF* and *PDE3B*; DL-P *versus* H: *DAB2*, *CD47* and *HLADRB4*; P *versus* H: *IGHDL-P*, *ITGB2* and *HLADRB4*. In conclusion, we identified that circulating lymphocytes and monocytes of individuals simultaneously affected by T2DM, dyslipidemia and periodontitis, showed an altered molecular profile mainly associated to inflammatory response, immune cell trafficking, and infectious disease pathways. Altogether, these results shed light on novel potential targets for future diagnosis, monitoring or development of targeted therapies for patients sharing these conditions.

## Introduction

Diabetes Mellitus (DM) is a metabolic disorder characterized by hyperglycemia as a consequence of defects in insulin secretion, in insulin’s mechanism of action or in both insulin’s secretion and action^1^. The onset of the disease is associated to genetic, environmental and/or behavioral risk factors^2^. Type 2 Diabetes Mellitus (T2DM) is a major public health problem that accounts for nearly 90% of all patients diagnosed with DM and is often associated with obesity and insulin resistance^3^. DM frequently occurs in synergy or concomitant with other systemic imbalances, such as dyslipidemia, a metabolic dysfunction that results from an increased level of lipoproteins in the blood^4^. Dyslipidemia (DL) could be one of the factors associated with DM-induced immune cell alterations^5^. However, even when blood glucose levels are well controlled, diabetic patients have a propensity to elevated low-density lipoprotein/triglycerides (LDL/TRG)^4–6^.

Another well-known risk factor for DM is periodontitis, a complex inflammatory disease that leads to loss of tooth support through periodontal and alveolar bone loss^7^. Although dental biofilm is the main etiologic factor in periodontitis, the activation of inflammatory mediators by the host in response to bacterial endotoxins are essential for disease progression. In response to this microbial challenge, periodontal inflammation promotes local and systemic elevations of pro-inflammatory cytokines, such as tumor necrosis factor-alpha (TNF-α), interleukin (IL)-1 beta (IL-1β) and IL-6^8^, as well as proteolytic enzymes and reactive oxygen species, which as a result produce alterations in the metabolism of lipids leading to dyslipidemia^4,5^. Elevated cytokine levels in turn lead to increased mobilization of lipids from the liver and adipose tissue^9^, raising the binding of low-density lipoprotein (LDL) to the endothelium and to smooth muscles, as well as the transcription of the LDL-receptor gene^6,10^.

Due to the bidirectional nature of periodontal disease and diabetes mellitus, and relationship between periodontitis and impaired lipid metabolism, it is common to find individuals affected by a combination of T2DM, dyslipidemia and periodontitis^6,11–13^. We have recently shown through gene expression analysis of *IL-10*, interferon-alpha and –gamma pathways in patients clinically selected based upon such pathological conditions, that dyslipidemia could be the leading disease that is associated with immune-related genes expression^14^.

In subsequent studies, we employed a primary screen by microarray (Human Genome U133) followed by an independent validation using RT-qPCR to identify potential genes related to poorly, or well-controlled T2DM individuals also affected by dyslipidemia and periodontitis, in comparison with patients without T2DM but affected by dyslipidemia and periodontitis^15^. Interestingly, we observed that poor glycemic control influenced a systemic exacerbation of gene expression related to immune response, as well as of genes associated with lipid metabolism and DNA replication/repair in peripheral blood mononuclear cells (PBMCs)^15^. Our findings led to further investigate the patients (i) without T2DM and dyslipidemia (systemic healthy) but affected only by periodontitis compared to (ii) individuals without any of these diseases (healthy control group) and we hypothesized that we would find a transcriptome signature specific for systemic healthy patients with periodontitis. The first additional group included in this study is the control for the T2DM and dyslipidemia, while the second additional group is the control for the systemic and oral investigated pathologies. As a result, we included the microarray analysis^15^ (considering of these additional groups); the transcriptome of each specific test group was compared with the healthy control group, and these results were submitted to functional enrichment analyses. In the present study, we aimed to identify the gene expression signature of circulating lymphocytes and monocytes from individuals simultaneously affected by T2DM (poorly or well-controlled), dyslipidemia and periodontitis, and other groups of patients presenting at least one of these complex diseases, compared to healthy individuals.

We investigated the gene expression signature of peripheral blood mononuclear cells (PBMCs) given that (i) it is a powerful approach for analysis of host responses during infection^16,17^ and (ii) an alternative to serum protein biomarkers to be used as a diagnostic tool to study disease pathogenesis and severity of cardiovascular, autoimmune and infectious diseases^17–20^. For instance, to better understand the pathobiology of periodontitis Papapanou *et al*.^21^ investigated monocytic gene expression signatures and assessed multiple serum inflammatory mediators, in order to correlate differences in the gene expression profile and the systemic inflammatory status of the patients. The authors also found that periodontal therapy may alter monocytic gene expression, which is consistent with a systemic anti-inflammatory effect. Moreover Kebschull and Papapanou (2010)^22^, reported in both, gingival and the circulating transcriptomes, a correlation between these transcriptomes with discernible phenotypic characteristics, also allowing an enhanced understanding of the pathobiology of the periodontal diseases and informing the design of subsequent studies. Finally, we have previously demonstrated that acute gingival inflammation may induce gene expression changes that modify tissue insulin/glucose metabolism^23^. And more recently we have reported that the levels of interleukins IL-1β and IL-37 in the gingival crevicular fluid (GCF) could reflect the local gingival tissue inflammatory response to the systemic, whole-body interactions demonstrating that circulating molecules may participate in the disease-related pathological molecular pathways^24^.

Our main goal in the present study was to identify specific gene expression signatures from circulating lymphocytes and monocytes to reveal potential biomarkers that may be used as molecular targets for future diagnosis of each combination of these pathologies (compared to healthy patients) and offer additional insight to the intricate underlying molecular mechanisms linking the pathways of each pathology.

## Results

### Sample population

Among the patients included in this study, no statistical differences were found between the participants regarding sex, ethnicity and socioeconomic status (Table 1). Groups including T2DM (T2DMpoorly-DL-P and T2DMwell-DL-P) had significantly higher levels of fasting glucose, HbA1c and HOMA index insulin resistance (Table 1). Insulin levels showed no significant differences between T2DMpoorly-DL-P and T2DMwell-DL-P groups^6^, as well as the BMI and abdominal circumference were similarly higher in the diabetic groups (T2DMpoorly-DL-P and T2DMwell-DL-P) compared with normoglycemic patients (H; Table 1). As expected, the total cholesterol, LDL, and triglyceride levels were higher in the T2DMpoorly-DL-P, T2DMwell-DL-P, and DL-P groups compared to P and H groups. Additional information can be found in our previous studies^14,25^.

**Table 1:** Demographic, physical, biochemical and periodontal characteristics of the patients (mean ± standard deviation).

	GROUP H n = 30	GROUP P n = 30	GROUP DL-P n = 30	GROUP T2DMwell-DL-P n = 30	GROUP T2DMpoorly-DL-P n = 30
Sex (F/M)	18/12	19/11	17/13	20/10	18/12
Age (mean ± SD)*	39.3 (+3.6)	45.9 (+5.9)^a^	49.0 (+7.5)^a^	50.3 (+6.7)^a^	48.0 (±7.6)^a^
Fasting glucose (mg/dl)*	85.9 (±6.5)	90.8 (±7.3)	90.0 (±6.4)	137.5 (±41.4)^b,c^	226.6 (±74.2)^a,b,c,d^
HbA_1c_ (%)*	5.4 (±0.21)	5.1 (±0.6)	5.4 (±0.6)	6.3 (±0.6)^a,b,c^	10.4 (±1.9)^a,b,c,d^
Insulin (U/L)*	7.1 (±4.3)	11.1 (±12.7)	12.6 (±8.5)	21.1 (±21.5)^a,b^	19.7 (±20.9)^a,b^
HOMA*	1.6 (±1.0)	2.9 (±3.5)	2.6 (±1.8)	6.8 (±5.2) ^a,b,c^	12.7(±15.9) ^a,b,c^
BMI (m/Kg^2^)*	24.5 (±3.5)	23.7 (±6.4)	28.4 (±3.8)	31.4 (±4.1)^a,b^	30.5 (±5.2)^a,b^
Abdominal circumference (cm)*	87.5 (±10.6)	98.2 (±16.9)	98.1 (±9.9)^a^	109.3 (±10.8)^a,b,c^	104.3 (±14.6)^a^
Total cholesterol (mg/dl)*	180.3 (±21.5)	171.6 (±18.5)	246.1 (±42.3)^a,b^	243.4 (±42.9)^a,b^	242.7 (±37.8)^a,b^
HDL cholesterol (mg/dl)*	49.3 (±10.1)	48.4 (±12.6)	50.7 (±11.1)	46.1 (±10.5)	44.8 (±9.5)
LDL cholesterol (mg/dl)*	113.5 (±18.1)	103.8 (±17.4)	156.4 (±44.1)^a,b^	147.3 (±44.3)^a,b^	153.4 (±37.0)^a,b^
Triglycerides (mg/dl)*	87.4 (±27.6)	93.9 (±35.9)	194.1 (±80.6)^a,b^	249.8 (±104.1)^a,b^	216.9 (±94.6)^a,b^
Number of teeth	27.1 (+1.8)	24.3 (+3.1)^a^	23.2 (+3.8)^a^	21.6 (+4.5)^a^	22.3 (+4.2)^a^
Percentage of sites with Bleeding on Probing	12.7 (+5.6)	51.4 (+13.2)^a^	53.0 (+13.7)^a^	53.9 (+13.8)^a^	69.3 (+12.8)^a,b,c,d^
Percentage of sites with Probing Depth ≤ 3 mm	98.8 (+1.5)	53.3 (+12.5)^a^	61.9 (+14.0)^a^	57.0 (+15.0)^a^	43.3 (+14.8)^a,b,c,d^
Percentage of sites with Probing Depth = 4–5 mm	1.2 (+1.5)	41.0 (+10.0)^a^	31.0 (+10.6)^a,b^	31.0 (+11.0)^a,b^	31.9 (+11.6)^a,b^
Percentage of sites with Probing Depth ≥ 6 mm	0.0 (+0.0)	5.7 (+5.9)^a^	7.0 (+10.6)^a^	12.0 (+10.8)^a,c^	24.8 (+15.9)^a,b,c,d^
Percentage of sites with Attachment Loss ≥ 5 mm	0.0 (+0.0)	28.4 (+10.5)^a^	23.8 (+14.0)^a^	34.7 (+17.6)^a,c^	47.1 (+16.2)^a,b,c,d^
Number of sites with Suppuration	0.0 (+0.0)	2.0 (+3.7)	2.0 (+3.0)	4.0 (+3.3)^a^	6.8 (+7.0)^a,b,c,d^

^a^p < 0.05 in relation to H (Healthy) group; ^b^p < 0.05 in relation to P (Periodontitis) group; ^c^p < 0.05 in relation to DL-P (Dyslipidemia-Periodontitis) group; ^d^p < 0.05 in relation to T2DMwell-DL-P (well-controlled-Type 2 Diabetes Mellitus-Dyslipidemia-Periodontitis) group according to the Kruskal-Wallis test, and Dunn’s post test; *α = 0.0125 (since Bonferroni’s correction = 0.05/4, i.e. four comparisons). Some data were also presented in Corbi *et al*.^16^ and Nepomuceno *et al*.^14^.

T2DM patients (T2DMpoorly-DL-P and T2DMwell-DL-P) presented P in a moderate to severe level, since they showed 4–5 mm of probing depth in ≥20% of the periodontal sites; 3–4 mm of clinical attachment loss in ≥30% of the periodontal sites, and ≥40% of the periodontal sites with bleeding on probing^14,25^. Both groups demonstrated similar periodontal tissue destruction, with bone loss and local inflammation. The T2DMwell-DL-P group showed a significant difference regarding the presence of deeper periodontal sites in comparison to groups without T2DM (DL-P, P and H)^6^. Patients from the T2DMpoorly-DL-P and T2DMwell-DL-P groups presented worse periodontal clinical condition than the P and DL-P^14,25^ (Table 1).

### Gene expression analysis and functional enrichment analyses by Ingenuity Pathway Analysis (IPA) and Gene Set Enrichment Analysis (GSEA)

In the circulating lymphocytes and monocytes gene expression analysis, when considering the T2DMpoorly-DL-P *versus* H comparison, we identified 1374 up- and down-regulated DEGs, while 869 were identified in the T2DMwell-DL-P *versus* H, 521 up- and down-regulated DEGs (DL-P *versus* H) and 564 up- and down-regulated DEGs (H *versus* P). The segregation of these pairwise comparisons of subjects by hierarchical cluster analysis is shown in Supplementary Figure 1.

**Figure 1:**
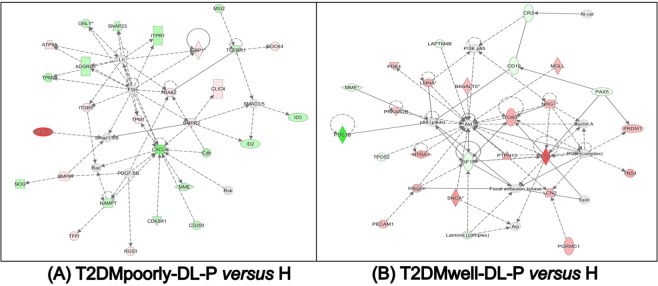
Ingenuity pathway analysis (IPA) of circulating lymphocytes and monocytes of the T2DMpoorly-DL-P *versus* H (**A**) and T2DMwell-DL-P *versus* H (**B**) study subjects. Functions and scores of 5 activated networks in this group comparison are listed in Table 2. Straight lines represent direct interaction, and dotted lines represent indirect interaction.

**Figure 2:**
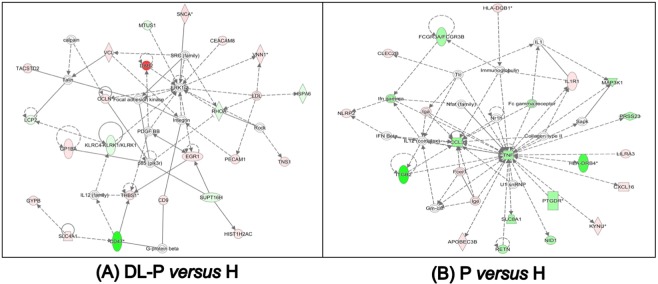
Ingenuity pathway analysis (IPA) of circulating lymphocytes and monocytes of the DL-P *versus* H **(A)** and P *versus* H **(B)** study subjects. Functions and scores of 5 activated networks in this group comparison are listed in Table 2. Straight lines represent direct interaction, and dotted lines represent indirect interaction.

To get a better insight into the biological function of the genes regulated by these inflammatory diseases, we performed Ingenuity Pathway Analysis (IPA) on all differentially expressed genes. Table 2 summarizes the networks identified in each pairwise group comparison, while the Top Canonical Pathways are found in the Supplementary Table 1. Noteworthy, Table 2 shows repeatedly networks associated with immunological pathways. Some of these enriched networks are illustrated in Figs. 1 and 2.

**Table 2:** Top Networks identified in circulating lymphocytes and monocytes by Ingenuity Pathway Analysis (IPA) in each pairwise comparison of the patients groups.

Pairwise comparison	Associated Network Functions	Score
T2DMpoorly-DL-P *versus* H	Cell Cycle, Cell-To-Cell Signaling and Interaction, Hematological System Development and Function	33
	Cellular Movement, Immune Cell Trafficking, Connective Tissue Disorders	31
	Antimicrobial Response, Inflammatory Response, Dermatological Diseases and Conditions	31
	Cellular Movement, Cellular Development, Cellular Growth and Proliferation	31
	Cellular Movement, Cancer, Organismal Injury and Abnormalities	29
T2DMwell-DL-P *versus* H	Cellular Movement, Immune Cell Trafficking, Cell-mediated Immune Response	37
	Infectious Diseases, Immunological Disease, Connective Tissue Disorders	33
	Hematological System Development and Function, Tissue Morphology, Cell-To-Cell Signaling and Interaction	33
	Cardiovascular System Development and Function, Tissue Morphology, Digestive System Development and Function	29
	Cell Death and Survival, Inflammatory Response, Organismal Injury and Abnormalities	27
DL-P *versus* H	Cellular Movement, Immune Cell Trafficking, Cell-To-Cell Signaling and Interaction	37
	Cellular Assembly and Organization, Cancer, Organismal Injury and Abnormalities	33
	Dermatological Diseases and Conditions, Developmental Disorder, Hereditary Disorder	33
	Cell-To-Cell Signaling and Interaction, Cell Morphology, Cellular Movement	31
	Organismal Development, Cardiovascular System Development and Function, Hematological System Development and Function	20
P *versus* H	Cell Death and Survival, Cell Signaling, Post-Translational Modification	34
	Dermatological Diseases and Conditions, Organismal Injury and Abnormalities, Cancer	34
	Inflammatory Disease, Inflammatory Response, Organismal Injury and Abnormalities	28
	Cardiac Arrhythmia, Cardiovascular Disease, Organismal Injury and Abnormalities	28
	Cellular Compromise, Inflammatory Response, Infectious Diseases	23

**Figure 3:**
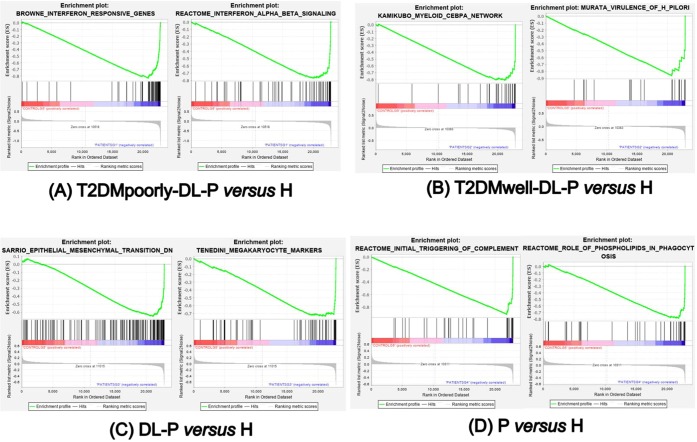
Gene sets of circulating lymphocytes and monocytes enriched in each comparison of each disease group *versus* healthy control group. Plots obtained by Gene Set Enrichment Analysis – GSEA (software version 4.0.3, http://www.broad.mit.edu/gsea). Enrichment scores and additional enriched gene sets are showed in Table 3.

We also performed the gene set enrichment analysis (GSEA) in the same group comparisons as in the IPA analysis. The top 20 enriched (curated) gene sets in the circulating lymphocytes and monocytes of each group comparison by GSEA are shown in the Table 3. In both comparisons, T2DMwell-DL-P vs. H and DL-P vs. H) there were fewer than 20 statistically significant enriched gene sets. In Table 3 we highlighted in gray the gene sets that are unique in each group comparison. From these unique gene sets, we selected in bold the gene sets that included genes enriched in the disease groups that were also found in the top IPA networks (Table 2). For example, in Table 3 the two selected gene sets (bold and gray highlighted) that are related to interferon responsive genes and interferon alpha and beta signaling genes where also found in the lists of the top IPA networks. The plots of those selected gene sets are shown in Fig. 3.

**Table 3:** Top 20 curated enriched gene sets in circulating lymphocytes and monocytes of each group comparison by GSEA (p < 0.05, FDR q-value < 0.05 and FWER p-value < 0.05).

T2DMpoorly-DL-P vs Healthy							
	GS follow link to MSigDB	SIZE	ES	NES	NOM p-val	FDR q-val	FWER p-val
1	JISON_SICKLE_CELL_DISEASE_UP	177	−1	−2.36	0	0	0
2	RAGHAVACHARI_PLATELET_SPECIFIC_GENES	69	−1	−2.27	0	0	0
3	REACTOME_ANTIMICROBIAL_PEPTIDES	72	−1	−2.19	0	0	0
4	**BROWNE_INTERFERON_RESPONSIVE_GENES**	**65**	**−1**	**−2.17**	**0**	**0**	**0**
5	TAKEDA_TARGETS_OF_NUP98_HOXA9_FUSION_3D_UP	180	−1	−2.12	0	0	0
6	MOSERLE_IFNA_RESPONSE	30	−1	−2.11	0	0	0
7	WIERENGA_STAT5A_TARGETS_DN	187	−1	−2.11	0	0	0
8	HECKER_IFNB1_TARGETS	90	−1	−2.11	0	0	0
9	BOSCO_INTERFERON_INDUCED_ANTIVIRAL_MODULE	73	−1	−2.08	0	0	0
10	REACTOME_NEUTROPHIL_DEGRANULATION	453	−1	−2.07	0	0	0
11	TAKEDA_TARGETS_OF_NUP98_HOXA9_FUSION_10D_UP	190	−1	−2.05	0	0	0
12	TONKS_TARGETS_OF_RUNX1_RUNX1T1_FUSION_ERYTHROCYTE_UP	157	−1	−2.04	0	0	0
13	REACTOME_DEFENSINS	29	−1	−2.04	0	0	0
14	BENNETT_SYSTEMIC_LUPUS_ERYTHEMATOSUS	27	−1	−2.03	0	0	0
15	HAHTOLA_SEZARY_SYNDROM_UP	94	−1	−2.03	0	0	0
16	SEITZ_NEOPLASTIC_TRANSFORMATION_BY_8P_DELETION_UP	72	−1	−2.02	0	0	0
17	**REACTOME_INTERFERON_ALPHA_BETA_SIGNALING**	**63**	−1	**−2.02**	**0**	**0**	**0**
18	SANA_RESPONSE_TO_IFNG_UP	70	−1	−2	0	0	0
19	TAKEDA_TARGETS_OF_NUP98_HOXA9_FUSION_16D_DN	121	−1	−2	0	0	0
20	DER_IFN_ALPHA_RESPONSE_UP	69	−1	−2	0	0	0
**T2DMwell-DL-P vs Healthy**							
	**GS follow link to MSigDB**	**SIZE**	**ES**	**NES**	**NOM p-val**	**FDR q-val**	**FWER p-val**
1	RAGHAVACHARI_PLATELET_SPECIFIC_GENES	69	−1	−2.333	0	0	0
2	REACTOME_ANTIMICROBIAL_PEPTIDES	72	−1	−2.16	0	0	0
3	PYEON_CANCER_HEAD_AND_NECK_VS_CERVICAL_DN	28	−1	−2.018	0	0	0
4	REACTOME_RESPONSE_TO_ELEVATED_PLATELET_CYTOSOLIC_CA2PLUS	128	−1	−1.986	0	0	0
5	COWLING_MYCN_TARGETS	41	−1	−1.978	0	0	0
6	JISON_SICKLE_CELL_DISEASE_UP	177	−1	−1.977	0	0	0
7	RICKMAN_HEAD_AND_NECK_CANCER_B	52	−1	−1.909	0	0.0034737	0.019
8	REACTOME_NEUTROPHIL_DEGRANULATION	453	−1	−1.892	0	0.0059291	0.037
9	**MURATA_VIRULENCE_OF_H_PILORI**	**21**	−1	−1**.886**	**0**	**0.0068948**	**0.049**
10	**KAMIKUBO_MYELOID_CEBPA_NETWORK**	**30**	−1	−1**.885**	**0**	**0.0063283**	**0.049**
**DL-P vs Healthy**							
	**GS follow link to MSigDB**	**SIZE**	**ES**	**NES**	**NOM p-val**	**FDR q-val**	**FWER p-val**
1	RAGHAVACHARI_PLATELET_SPECIFIC_GENES	69	−1	−2.394	0	0	0
2	WIERENGA_STAT5A_TARGETS_DN	187	−1	−2.276	0	0	0
3	PYEON_CANCER_HEAD_AND_NECK_VS_CERVICAL_DN	28	−1	−2.204	0	0	0
4	REACTOME_ANTIMICROBIAL_PEPTIDES	72	−1	−2.166	0	0	0
5	VALK_AML_CLUSTER_8	25	−1	−2.143	0	0	0
6	GNATENKO_PLATELET_SIGNATURE	42	−1	−2.123	0	0	0
7	RICKMAN_HEAD_AND_NECK_CANCER_B	52	−1	−2.108	0	0	0
8	REACTOME_RUNX1_REGULATES_GENES_INVOLVED_IN_MEGAKARYOCYTE_DIFFERENTIATION_AND_PLATELET_FUNCTION	78	−1	−2.06	0	3.22E-04	0.002
9	HAHTOLA_SEZARY_SYNDROM_UP	94	−1	−2.059	0	2.86E-04	0.002
10	REACTOME_RESPONSE_TO_ELEVATED_PLATELET_CYTOSOLIC_CA2PLUS	128	−1	−2.043	0	3.85E-04	0.003
11	**TENEDINI_MEGAKARYOCYTE_MARKERS**	**61**	−1	**−2.034**	**0**	**4.68E-04**	**0.004**
12	JAATINEN_HEMATOPOIETIC_STEM_CELL_DN	228	−1	−2.009	0	8.62E-04	0.008
13	REACTOME_NEUTROPHIL_DEGRANULATION	453	−1	−2.002	0	8.96E-04	0.009
14	REACTOME_AMYLOID_FIBER_FORMATION	89	−1	−1.989	0	0.0011131	0.011
15	TAKEDA_TARGETS_OF_NUP98_HOXA9_FUSION_16D_DN	121	−1	−1.988	0	0.0010389	0.011
16	**SARRIO_EPITHELIAL_MESENCHYMAL_TRANSITION_DN**	**144**	−1	−1**.985**	**0**	**9.74E-04**	**0.011**
17	VALK_AML_CLUSTER_7	27	−1	−1.977	0	0.0011464	0.014
18	JISON_SICKLE_CELL_DISEASE_UP	177	−1	−1.962	0	0.0017301	0.022
**Periodontitis vs Healthy**							
	**GS follow link to MSigDB**	**SIZE**	**ES**	**NES**	**NOM p-val**	**FDR q-val**	**FWER p-val**
1	**REACTOME_INITIAL_TRIGGERING_OF_COMPLEMENT**	**39**	−1	**−2.334**	**0**	**0**	**0**
2	REACTOME_CREATION_OF_C4_AND_C2_ACTIVATORS	32	−1	−2.256	0	0	0
3	FARMER_BREAST_CANCER_CLUSTER_1	45	−1	−2.242	0	0	0
4	REACTOME_COMPLEMENT_CASCADE	71	−1	−2.222	0	0	0
5	REACTOME_SCAVENGING_OF_HEME_FROM_PLASMA	30	−1	−2.221	0	0	0
6	REACTOME_FCERI_MEDIATED_CAPLUS2_MOBILIZATION	48	−1	−2.151	0	0	0
7	JISON_SICKLE_CELL_DISEASE_UP	177	−1	−2.136	0	0	0
8	RAGHAVACHARI_PLATELET_SPECIFIC_GENES	69	−1	−2.127	0	0	0
9	REACTOME_ROLE_OF_LAT2_NTAL_LAB_ON_CALCIUM_MOBILIZATION	33	−1	−2.116	0	0	0
10	REACTOME_FCERI_MEDIATED_MAPK_ACTIVATION	49	−1	−2.112	0	0	0
11	REACTOME_CONDENSATION_OF_PROPHASE_CHROMOSOMES	55	−1	−2.099	0	0	0
12	REACTOME_REGULATION_OF_ACTIN_DYNAMICS_FOR_PHAGOCYTIC_CUP_FORMATION	77	−1	−2.062	0	0	0
13	REACTOME_FCGR_ACTIVATION	30	−1	−2.058	0	0	0
14	REACTOME_ACTIVATED_PKN1_STIMULATES_TRANSCRIPTION_OF_AR_ANDROGEN_RECEPTOR_REGULATED_GENES_KLK2_AND_KLK3	48	−1	−2.052	0	0	0
15	REACTOME_AMYLOID_FIBER_FORMATION	89	−1	−2.047	0	0	0
16	REACTOME_HDACS_DEACETYLATE_HISTONES	71	−1	−2.033	0	0	0
17	WALLACE_PROSTATE_CANCER_RACE_UP	289	−1	−2.024	0	0	0
18	**REACTOME_ROLE_OF_PHOSPHOLIPIDS_IN_PHAGOCYTOSIS**	**42**	−1	**−2.02**	**0**	**0**	**0**
19	REACTOME_PRC2_METHYLATES_HISTONES_AND_DNA	54	−1	−2.012	0	0	0
20	REACTOME_DNA_METHYLATION	46	−1	−2.012	0	0	0

**Figure 4:**
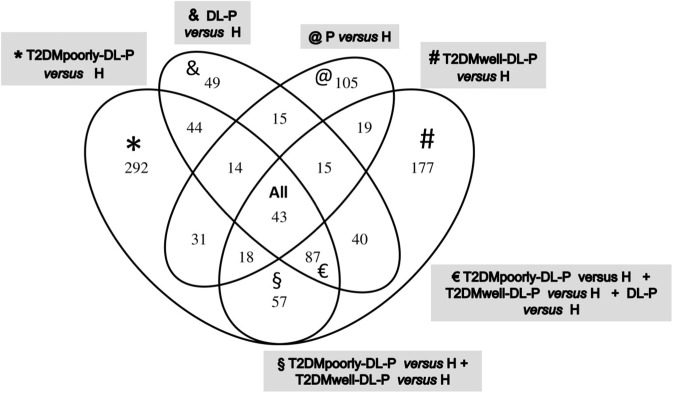
Venn diagram of the up-regulated genes from circulating lymphocytes and monocytes of each group comparison. A list of selected genes from each group can be found in Table 4.

The Venn diagram in Fig. 4 exhibits the up-regulated differentially expressed genes that were unique in circulating lymphocytes and monocytes of each disease group compared to the healthy control group. Analyzing the Fig. 4 in combination with the Table 4, we observed that the comparison of T2DMwell-DL-P vs. H revealed 177 up-regulated genes. From those 177 up-regulated genes, 17 were related to immune response. Table 4 shows that in the first three group comparisons, as the metabolic impairment of the patient decreases, the number of up-regulated genes related with the immune response also decreases.

**Table 4:** Top Up-regulated Gene Ontology Biological Processes and selected genes found in circulating lymphocytes and monocytes of each group comparison.

Group Comparison	Top Up-regulated Gene Ontology Biological Process	Number of genes in this category	Represented genes found only in this comparison (Name/ Function)
* T2DMpoorly-DL-P *versus* H	immune response/ chemotaxis/ inflammatory response/innate immune response/ apoptosis / caspase activation/	37	CXCL10 (chemokine (C-X-C motif) ligand 10), IL7 (Interleukin 7), CCR1 (chemokine (C-C motif) receptor 1), NLRC4 (NLR family, CARD domain containing 4/ interleukin-1 beta secretion) SERPING1 (serpin peptidase inhibitor, clade G (C1 inhibitor), member 1/ innate immune response), OAS1, OAS3 (2’,5’-oligoadenylate synthetase 1; and 3), GBP1 (guanylate binding protein 1, interferon-inducible)
	signal transduction/defense response /response to virus	11	STAT1 (signal transducer and activator of transcription 1), MX1 (Interferon-Inducible Protein P78), IFI6 (interferon, alpha-inducible protein 6), RSAD2 (radical S-adenosyl methionine domain containing 2)
	glucose/ carbohydrate/ lipid metabolic processes	8	INSIG1 (insulin induced gene 1), FKFB3 (6-phosphofructo-2-kinase/fructose-2,6-biphosphatase 3), ACSL1 (acyl-CoA synthetase long-chain family member 1), GBA (glucosidase, beta; acid)
	metabolic processes	5	DHRS9 (dehydrogenase/reductase (SDR family) member 9), ATP9A (ATPase, class II, type 9A), HGD (homogentisate 1,2-dioxygenase)
# T2DMwell-DL-P *versus* H	innate immune response/ immune response/ defense response/ apoptosis / inflammatory response	17	CLEC12A; CLEC12B (C-type lectin domain family 12, member A; member B), ITGB1 (integrin, beta 1), SERGN (serglycin/platelet degranulation/ negative regulation of cytokine secretion), IL2RA (interleukin 2 receptor, alpha), ELANE (elastase 2, neutrophil)
	glucose/ lipid/ metabolic processes	6	PFKP (phosphofructokinase, platelet), CPT1A (carnitine palmitoyltransferase 1A (liver), PLCB1 (phospholipase C, beta 1)
	metabolic processes	4	ENPP4 (ectonucleotide pyrophosphatase/phosphodiesterase 4), CEBPD (CCAAT/enhancer binding protein (C/EBP), delta), SMAD5 (SMAD family member 5)
& DL-P versus H	immune response	2	IL1R2 (interleukin 1 receptor, type II), GBP5 (guanylate binding protein 5)
	glucose/ lipid/ metabolic processes	2	AKR1C3 (aldo-keto reductase family 1, member C3), PTGDS (prostaglandin D2 synthase)
@ P *versus* H	innate immune response/ immune response/ chemotaxis/ defense response	10	FTH1 (ferritin, heavy polypeptide 1), FCGR2B (Fc fragment of IgG, low affinity IIb, receptor (CD32)), CXCL16 (chemokine (C-X-C motif) ligand 16), NLRP2 (NLR family, pyrin domain containing 2)
	carbohydrate metabolic processes	3	GK (glycerol kinase), ST8SIA1 (ST8 alpha-N-acetyl-neuraminide alpha-2,8-sialyltransferase 1), B3GALTL (beta 1,3-galactosyltransferase-like),
	metabolic processes/ signaling pathway	8	SEL1L (SEL1L Adaptor Subunit Of ERAD E3 Ubiquitin Ligase), DUSP2 (dual specificity phosphatase 2), KYNU (kynureninase (L-kynurenine hydrolase)
§ T2DMpoorly-DL-P *versus* H + T2DMwell-DL-P *versus* H (T2DM groups, independent of glycemic control)	innate immune system/ chemotaxis/	7	CTSL1 (cathepsin L1), RETN (resistin), CLU (clusterin), OASL (2’−5’-oligoadenylate synthetase-like), CXCL5 (chemokine (C-X-C motif) ligand 5), CRISP3 (cysteine-rich secretory protein 30, FCGR1A (Fc fragment of IgG, high affinity Ia, receptor (CD64)
	lipid metabolic processes	1	FAR2 (Fatty Acyl-CoA Reductase 2)
	metabolic processes	7	MMP9 (matrix metallopeptidase 9), MMP8 (matrix metallopeptidase 8 -neutrophil collagenase), ARG1 (arginase, liver), B4GALT5 (Beta-1,4-Galactosyltransferase 5)
€ T2DMpoorly-DL-P versus H + T2DMwell-DL-P versus H + DL-P *versus* H (DL-P groups)	defense response/ immune response/ innate immune response/ apoptosis	10	DEFA1, DEFA3, DEFA4 (defensin, alpha 1; alpha 3; alpha 4), MPO (myeloperoxidase), BPI (bactericidal/permeability-increasing protein), CAMP (cathelicidin antimicrobial peptide)
	carbohydrate and lipid metabolic processes	6	PTGS1 (prostaglandin-endoperoxide synthase 1 (prostaglandin G/H synthase and cyclooxygenase), NRG1 (neuregulin 1), BPGM (2,3-bisphosphoglycerate mutase), PDK4 (pyruvate dehydrogenase kinase, isozyme 4), MGLL (monoglyceride lipase)
	protein amino acid phosphorylation/ signaling transduction processes	7	MYLK (myosin light chain kinase),PRKAR2B (protein kinase, cAMP-dependent, regulatory, type II, beta), RHOBTB1 (Rho-related BTB domain containing 1), PDE5A (phosphodiesterase 5 A, cGMP-specific)
All comparisons	defense response/ immune response	3	CLEC4D (C-type lectin domain family 4, member D), LTF (lactotransferrin)
	lipid biosynthetic process	1	ELOVL7 (ELOVL family member 7, elongation of long chain fatty acids)
	metabolic processes	5	ALAS2 (aminolevulinate, delta-, synthase 20), ENOSF1 (enolase superfamily member 1)

Symbols refer to the group comparisons showed in the Fig. 4 (Venn diagram). Example: * In the T2DMpoorly-DL-P *versus* H were found 292 genes up-regulated, being 37 of them belonging to the immune system (Number of genes in this category of the Gene Ontology Biological Processes), in which some of the selected genes were mentioned.

### Validation by RT-qPCR of selected genes from the network analysis

**Figure 5:**
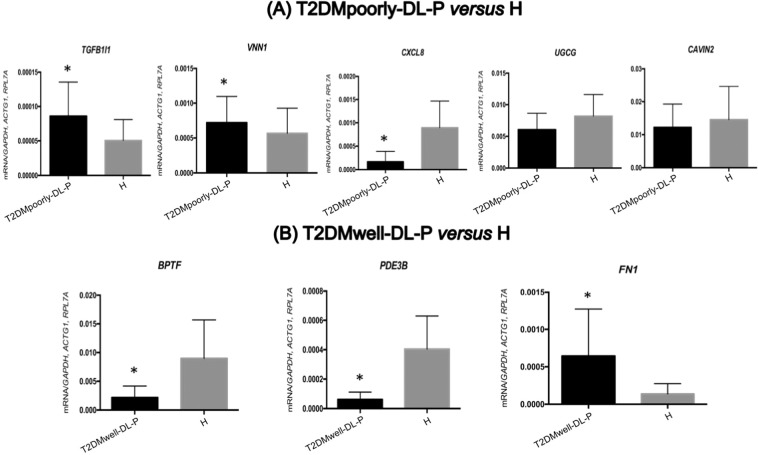
Validation results of circulating lymphocytes and monocytes by RT-qPCR in T2DMpoorly-DL-P *versus* H **(A)** and T2DMwell-DL-P *versus* H **(B)**. All mRNA levels of the investigated genes were normalized to a mean of the endogenous controls *GAPDH*, *ACTG1* and *RPL7A* genes. Data represent the mean ± SD of 30 patients per group (Mann-Whitney U test; α = 5%). *Significant *p values* compared to H. (**A**) *TGFB1I1* gene, *p values* = 0.0115; *VNN1* gene, *p values* = 0.0467; *CXCL8* gene, *p values* <0.0001; *UGCG* gene, *p values* = 0.0392 and *CAVIN2* gene, *p values* = 0.2928. (B) *BPTF* gene, *p values* <0.0001; *PDE3B* gene, *p values* <0.0001; *FN1* gene, *p values* <0.0001.

**Figure 6:**
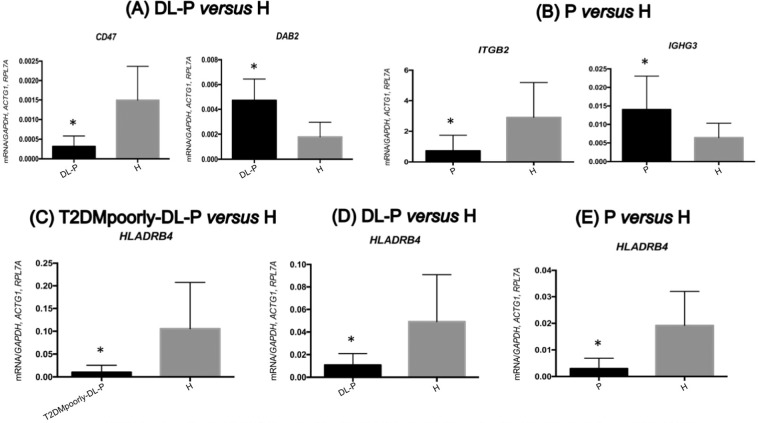
Validation results of circulating lymphocytes and monocytes by RT-qPCR in DL-P *versus* H **(A)**, P *versus* H **(B)**, T2DMpoorly-DL-P *versus* H **(C)**, DL-P *versus* H **(D)** and P *versus* H **(E)**. All mRNA levels of the investigated genes were normalized to a mean of the endogenous controls *GAPDH*, *ACTG1* and *RPL7A* genes. Data represent the mean ± SD of 30 patients per group (Mann-Whitney U test; α = 5%). *significant *p values* compared to H. (**A**) *CD47* gene, *p values* <0.0001; *DAB2* gene, *p values* <0.0001. (**B**) *ITGB2* gene, *p values* <0.0001; *IGHDL-P* gene, *p values* = 0.0053. (**C**) *HLADRB4* gene, *p values* <0.0001. (**D**) *HLADRB4* gene, *p values* = 0.0018. (**E**) *HLADRB4* gene, *p values* = 0.0002.

Among the circulating lymphocytes and monocytes top up- and down-regulated genes present in the enriched biological pathways, we selected three to five genes from each disease group to validate by RT-qPCR. The results are shown in Figs. 5 and 6. The results of RT-qPCR comparing T2DMpoorly-DL-P *vs*. H, which assessed the influence of poor glycemic control of T2DM added to the presence of dyslipidemia and periodontitis, validated the importance of the *TGFB1I1* (transforming growth factor beta 1 induced transcript 1) and the *VNN1* (vanin 1) genes, since they were upregulated on T2DMpoorly-DL-P subjects, while the *HLADRB4* (major histocompatibility complex, class II, DR beta 4) and the *CXCL8* (C-X-C motif chemokine ligand 8) genes were downregulated in the same group (Figs. 5A and 6C).

On the T2DMwell-DL-P *vs*. H comparison, which evaluated the stimulus of T2DM with good glycemic control and the presence of dyslipidemia and periodontitis, the RT-qPCR results from circulating lymphocytes and monocytes validated that the *BPTF* (bromodomain PHD finger transcription factor) and *PDE3B* (phosphodiesterase 3B) genes were upregulated in H, and the *FN1 (Fibronectin 1)* gene was downregulated in the same group (Fig. 5B).

On the DL-P *vs*. H comparison, which evaluated the effect of the dyslipidemia and periodontitis, the qPCR confirmed that the *HLADRB4* and *CD47* (CD47 molecule) genes were upregulated in H, while the *DAB2* (DAB adaptor protein 2) gene was downregulated in H (Fig. 6A,D). Lastly, the P *vs*. H comparison, which evaluated the association with periodontitis, the RT-qPCR from circulating lymphocytes and monocytes confirmed that the *HLADRB4* and *ITGB2* (integrin subunit beta 2) genes were upregulated in H, while the *IGHDL-P* (immunoglobulin heavy constant gamma 3) gene was downregulated in H (Fig. 6B,E).

## Discussion

To the best of our knowledge, this is the first study describing functional information related to the circulating lymphocytes and monocytes transcriptional profile of individuals affected (simultaneously or not) by three of the currently most prevalent diseases: T2DM, dyslipidemia and periodontitis. Using a gene expression dataset generated from PBMC followed by functional enrichment analyses, we showed that validated DEGs were implicated mainly in immune cell trafficking, antimicrobial and inflammatory response, cell-to-cell signaling and interaction, infectious and cardiovascular diseases, cellular growth and proliferation.

When we evaluated the circulating lymphocytes and monocytes expression profiling of T2DMpoorly-DL-P *vs* H, we sought to find DEGs responsible for the influence of poor glycemic control of T2DM in the presence of dyslipidemia and periodontitis. The RT-qPCR results demonstrated that the *TGFB1I1* and *VNN1* genes were upregulated in T2DMpoorly-DL-P in comparison to H. Functional information of the *VNN1* gene can be found in the Supplementary Discussion section. Based on the functional network analysis of the microarray data, these genes are associated with cellular signaling and movement, cancer, molecular transport, vitamin/mineral metabolism, organism injury and abnormalities. Considering the upregulated genes in this pairwise comparison, the *TGFB1I1* gene encodes a secreted ligand of the TGF-beta (transforming growth factor-beta) superfamily of proteins. Ligands of this family bind various TGF-beta receptors leading to recruitment and activation of the SMAD family, which are signal transducers, and transcriptional modulators that mediate the TGF-beta signal and thus regulate multiple cellular processes^26^. The protein encoded by the *TGFB1I1* gene regulates cellular proliferation, differentiation and growth, and modulate the expression and activation of other growth factors including interferon gamma (IFN-γ) and tumor necrosis factor alpha (TNF-α)^26,27^. Our present results lead to the hypothesis that the circulating *TGFB1I1* overexpression in T2DMpoorly-DL-P subjects could contribute to an exaggerated cellular activation and immune response compared to individuals in H. To this date, we were unable to find previously published data showing the circulating lymphocytes and monocytes expression of this gene in the three pathologies studied here. Based on these findings, further studies should be designed and performed to validate and investigate the effects on peripheral cells and organs affected by these diseases. Otherwise, increased *TGFB1I1* mRNA expression level was identified in individuals exhibiting rheumatoid arthritis in comparison with individuals with osteoarthritis, both conditions presenting the fibroblast like synoviocytes^28^.

Interestingly, the *CXCL8 (C-X-C Motif Chemokine Ligand 8)* gene was downregulated in T2DMpoorly-DL-P compared to H subjects. The *CXCL8 gene*, previously named as interleukin 8 (*IL-8)*, encodes the protein that is a key chemokine in the initiation and amplification of acute inflammatory reactions and in chronic inflammatory processes because it attracts and activates neutrophils in inflammatory regions^29,30^. Regarding periodontitis, there is an interplay between microbial species within subgingival biofilms and the adjacent periodontal tissues^6^, which elicit the innate immunity by releasing chemokines, such as the CXCL-8/IL-8 into the gingival crevicular fluid (GCF)^31^ necessary to recruiting neutrophils. We hypothesize that there should be an equilibrated *CXCL8/IL8* mRNA and protein production to obtain adequate host immune response against periodontal pathogens driving to a healthy periodontal status. In spite to contradictory studies regarding the salivary IL-8 levels in periodontitis patients Khalaf *et al*.^32^ reported significantly higher IL-8 levels in saliva from periodontally healthy individuals in comparison with those affected by periodontitis. This result is in agreement with a meta-analysis that showed higher levels of IL-8 (pg/µL) in the GCF of periodontally healthy control subjects compared with periodontitis patients^33^. We also found that the circulating lymphocytes and monocytes *CXCL8/IL8* mRNA levels of periodontally healthy subjects were significantly lower when compared to patients affected concomitantly by T2DM, dyslipidemia and periodontitis. In addition, to the evidences of the systemic higher expression of pro-inflammatory cytokines, such as IL-1β, IL-6 and TNF-α in PBMC of T2DM patients^34–37^, our results suggest that the circulating lymphocytes and monocytes present a hyper-inflammatory state, together with lower levels of the *CXCL8/IL8* expression, might contribute to the disturbance in the orchestration of the immune response in patients affected by these diseases.

Interestingly, the IPA revealed in a unique network (Fig. 1A) the downregulation of *CXCL8/IL8* gene in T2DMpoorly-DL-P compared to H subjects contrasting with the upregulation of the *TGFB1I1* gene in the circulating lymphocytes and monocytes of the same subjects. To our knowledge, it is the first time that both *CXCL8/IL8* and *TGFB1I1* genes are found in blood peripheral cell of patients with this combination of diseases and significantly contrasting to each other. Obviously, since the mechanism by which this network might affect the characteristics of the investigated three diseases is largely unknown, further studies are needed to explain these findings and relationships. In addition, the transcriptomic analysis of T2DMwell-DL-P *versus* H, DL-P *versus* H, as well the P *versus* H, presented some validated genes (Figs. 5B and 6) which are discussed in the Supplementary Discussion section.

The findings of Fig. 6A that showed the validated downregulation of the *CD47* gene and upregulation of the *DAB2* gene in circulating lymphocytes and monocytes of DL-P patients are in agreement with the IPA analysis (Fig. 2A). These genes are correlated on the same network associated with cell-to-cell signaling and interaction, cell morphology and cellular movement. The IPA analysis also showed the validated *HLADRB4* and *ITGB2* genes both downregulated in Periodontitis patients (group P), interacting via TNF on the same network (Fig. 2B) of cellular compromise, inflammatory response, and infectious diseases. Finally, the *HLADRB4* gene was validated in peripheral blood of T2DMpoorly-DL-P, DL-P and P *versus* H comparisons, and was always found to be upregulated in H (systemic and oral healthy subjects) (Figs. 4E and 6C–D). The *HLA-DRB4 (Major Histocompatibility Complex, Class II, DR Beta 4)* encodes the HLA-DRB4 protein from the HLA class II beta chain paralogues, and this class II molecule is a heterodimer consisting of an alpha (DRA) and a beta (DRB) chain, both anchored in the membrane^38^. Class II molecules are expressed in antigen presenting cells (B lymphocytes, dendritic cells, macrophages)^39^, playing a central role in the immune system by presenting peptides derived from extracellular proteins^40^. Our present results lead to the hypothesis that the *HLADRB4* expression levels indicate the healthy status of the patients, since it was consistently upregulated in circulating lymphocytes and monocytes of systemically healthy individuals without periodontitis (H). Further studies will need to investigate a potential correlation between circulating high levels of *HLADRB4* and low levels of pro-inflammatory expression in individuals with some chronic inflammatory disease, such as poor metabolic controlled T2DM, dyslipidemia and periodontitis.

The Venn diagram (Fig. 4) shows the number of genes specifically upregulated in circulating lymphocytes and monocytes of each group comparisons. Deeper analyses of those findings, followed by further investigations to assess their validity and functionality, might be useful to indicate blood biomarkers for diagnosis of the combined pathologies, for monitoring those patients in treatment, or to provide insights into novel therapeutic strategies for disease prevention and treatment.

Enriched gene sets detected by the GSEA included genes enriched in the circulating lymphocytes and monocytes of diseased groups that were also find in the top IPA networks. Therefore, taking together the functional enrichment analyses by IPA and GSEA, we observed many similarities regarding biological functions identified for each group comparison, but the peripheral blood molecular signaling specificities demand deeper analyses, as well as additional *in vitro* and *in vivo* experiments. Comparing the Venn diagram and the gene ontology findings showed in Table 4, the comparison T2DMpoorly-DL-P vs. H group showed twice the number of up-regulated genes related with the immune response (37) in comparison to the 17 up-regulated genes in the same category in the T2DMwell-DL-P vs. H group. Even though both groups are affected by T2DM and it is recognized that these patients reflect a hyperinflammatory immune response state^41^, our results suggest that the patients with increased metabolic impairment can elicit the immune response in a broader and different way than the patients presenting adequate metabolic control. Nonetheless, the up-regulated group-specific genes expressed in circulating lymphocytes and monocytes as showed in the Venn diagram (Fig. 4) should be further investigated.

Despite the significant amount of novel data showed here, the main limitations of this study are (i) the transcriptome signature presented here is specific to circulating lymphocytes and monocytes; therefore the transcriptome signature of any organ or any other cell type (e.g. liver, pancreas, kidney, periodontium, neutrophils, etc) was not included. Consequently, the results showed here should be placed in the context of the circulating lymphocytes and monocytes, which in fact, was a main goal of our study. We acknowledge the limitations to discuss how the circulating lymphocytes and monocytes, and their molecular signature, may impact how these cells home and act in target tissues of T2DM, Dyslipidemia and Periodontitis; (ii) the lack of longitudinal data tracking the disease progression in our cohort of patients, and (iii) the absence of genetic analyses at the translational level. Further studies enrolling additional larger and ethnically diverse population of patients are important to search for external validation of the present findings. Moreover, the present study was not designed to assess whether the disease phenotype of each group of patients was the cause or consequence of the gene expression signature. Of importance, even though we believe that the PBMC transcriptome data herein presented is novel and relevant, we clearly acknowledge its limitations when compared to big-data approaches, such as the Schüssler -Fiorenza Rose *et al*.^42^, multi-omics studies including the microbiome such as the Zhou *et al*.^43^, or other large-scale dataset methodologies developed to integrate the transcriptome within the context of the interactome, similarly to Li *et al*.^44^, which developed a PBMC transcriptome analysis, and constructed an interactome to investigate the multi-layered regulatory pathways in T2DM.

In addition to big-data and host-microbiome interaction studies, methods such as transcriptome, proteome and interactome of other cell types could contribute to further elucidate and/or corroborate our findings. Therefore, since T2DM and dyslipidemia are complex multi-system diseases, our present PBMC transcriptome is able to demonstrate one layer of the multi-omics signatures that could be investigated. Certainly, further studies are important to reach the biological mechanisms mediating the association of the DEGs in PBMC with each specific pathological combination, preferably in a broader and deeper way, such as by using multi-omics strategies to understand the interactions of the different biological aspects of each combination of complex diseases.

Here we identified the differences in the circulating lymphocytes and monocytes expressed genes in patients with different combinations of T2DM, DL and P. We believe that our results represent an important advance in the field, and bring to light a diverse range of molecules in the PBMC that can be further investigated for their validity as biomarkers for a combination of pathologies as well as their potential functional role in the physiological and pathological environment. Strong aspects of this study include (i) the selected patients were subjected to strict clinical inclusion criteria accurately distinguishing the pathological condition between patients, permitting to identify specific PBMC DEGs related to each of these clinical conditions; (ii) the data was consistently validated for the PBMC expression levels by RT-qPCR method, demonstrating the internal validity of the study.

In conclusion, we identified and validated a gene expression signature for circulating lymphocytes and monocytes from T2DM (poorly or well-controlled), dyslipidemia and periodontitis patients and showed the most relevant functional pathways of each pathological combination. Additional studies are needed to externally validate our findings and evaluate whether these genes can in the future be used as peripheral blood markers for risk assessment of each complex disease combination, as well as can provide novel insights into targeted therapeutic strategies for disease prevention and/or treatment.

## Materials and Methods

### Study population

All experimental protocols were approved by the Ethics in Human Research Committee of School of Dentistry at Araraquara (UNESP – São Paulo State University, Araraquara, Brazil; Protocol number 50/06), and all the volunteers signed an informed consent. All methods were carried out in accordance with the principals of the Declaration of Helsinki.

From 2009 to 2011, we evaluated 1788 patients; the study’s inclusion criteria included age range from 35 to 60 years, presence of at least 15 natural teeth and similar socio-economic level^6^. We analyzed 150 patients who were divided into five groups of 30 patients each, based upon diabetic, dyslipidemic and periodontal status: poorly controlled T2DM with dyslipidemia and periodontitis (T2DMpoorly-DL-P); well-controlled T2DM with dyslipidemia and periodontitis (T2DMwell-DL-P); normoglycemic individuals with dyslipidemia and periodontitis (DL-P); systemically healthy individuals with periodontitis (P); and systemically healthy individuals without periodontitis (H)^14^. The exclusion criteria were previously described in Corbi *et al*.^25^.

### Physical, biochemical and periodontal evaluations

Each subject was submitted to physical examination including anthropometric data such as abdominal circumference (cm), hip (cm), waist (cm) and height (m), weight (kg), and body mass index (BMI)^6^.

Blood samples were collected after a 12-hour overnight fast for the evaluation of fasting plasma glucose (mg/dL) by modified Bondar & Mead method, glycated haemoglobin (HbA1c) by enzymatic immunoturbidimetry, insulin levels by the chemiluminescence method (U/L) and high-sensitivity C-reactive protein by the nephelomeric method^6^. Insulin resistance was evaluated by calculation of the homeostasis model assessment (HOMA). The same laboratory performed all analyses. Patients were considered as nondiabetics (normoglycemic individuals) if they presented fasting glucose levels <100 mg/dL and HbA1c < 6.5%. T2DM patients were diagnosed by an endocrinologist who monitored their glycemic levels by evaluation of HbA1c; patients were divided into poorly controlled (HbA1c ≥ 8.5%, 64 mmol/mol) or well-controlled patients (HbA1c < 7.0%)^14^. Diabetic individuals with HbA1c between 7.0–8.5% were not included.

Lipid profile [total cholesterol (TC), triglycerides (TGs), and HDL] was performed by enzymatic methods. LDL was determined by the Friedewald formula. To avoid the inclusion of individuals with transitory dyslipidemia, the cutoff points used here were the highest values according to the National Cholesterol Educational Program (NCEP) Adult Treatment III (ATP III)^45^: TC ≥ 240 mg/dL, LDL ≥ 160 mg/dL, HDL ≤ 40 mg/dL, and TGs ≥200 mg/dL^6^.

Diagnosis of periodontal disease as defined by the American Academy of Periodontology^46^ includes local signs of inflammation and tissue destruction (presence of deep periodontal pockets ≥6 mm) and loss of the connective tissue attachment of gingiva to teeth (clinical attachment loss ≥4 mm) in at least 4 non-adjacent teeth. All patients were subjected to a periodontal clinical examination performed in six sites per tooth by a single trained calibrated examiner (A.B.S., Kappa = 0.89) as described previously^6,14,25^. Periodontal pocket depth, clinical attachment loss, and bleeding on probing were evaluated with a periodontal probe PCP UNC 15 (Hu-Friedy®). Severe periodontitis criteria was defined as the presence of deep periodontal pockets ≥6 mm with clinical attachment loss ≥5 mm and bleeding on probing in at least 8 sites distributed in different quadrants of the dentition^40,25^.

### Microarray, RT-qPCR and statistical analysis

Total RNA was extracted from peripheral blood mononuclear cells (PBMCs) since we focused on the circulating lymphocytes and monocytes gene expression signature. After extraction and purification of RNA samples, only those in the λ(260/280) and λ(260/230) ratios between 1.8 to 2.2 were used. Details regarding the aforementioned methodologies can be found in the Supplementary Materials and Methods. Microarray data (U133 Plus 2.0, Affymetrix Inc., Santa Clara, CA, USA) was generated from patients T2DMpoorly-DL-P (n = 5), T2DMwell-DL-P (n = 7), DL-P (n = 6), P (n = 6) and H (n = 6), after considering greater homogeneity regarding biochemical, lipid and clinical periodontal parameters^6^.

The raw.CEL files were preprocessed using the Robust Multichip Average (RMA) strategy, as implemented in the Affy package^47^, which performs background correction through a normal-exponential convolution model, quantile normalizes the probe intensities and summarizes them into probeset-level quantities using an additive model fit through the median-polish strategy. The log2-expression quantities resulted from the RMA method were further processed by the RankProd package^48^. Probesets that presented $|{\log }_{2}FC| > 1$ and percentage of false prediction $(pfp) < 0.01$ were called up- or downregulated, depending on the sign of the log-FC. Pairwise comparisons were made between the H (systemically and oral healthy subjects) and each of the T2DMpoorly-DL-P, T2DMwell-DL-P, DL-P and P groups (T2DMpoorly-DL-P *versus* H; T2DMwell-DL-P *versus* H; DL-P *versus* H and P *versus* H). The significant probesets from the aforementioned workflow were analysed with the Ingenuity Pathway Analysis software (IPA; Build 470319 M, Version 43605602, Qiagen, Redwood City, CA) as well as the Gene Set Enrichment Analysis software (GSEA; version 4.0.3 http://www.broad.mit.edu/gsea). Additional methods details from processing and analyses can be found at the Supplementary Materials and Methods section.

Independent validation of selected DEGs was performed by RT-qPCR analysis in a total of 150 patients (n = 30 each group, including the patients chosen for microarray analysis). Statistical analyses were performed in the GraphPad Prism software (version 5.0) using a significance level of 0.05^6^.

## Supplementary information

Suplementary Information.

## Data Availability

The data that support the findings of this study are available from the corresponding author, RMSC, upon reasonable request.
