# Supplementary material for: Circulating lymphocytes and monocytes transcriptomic analysis of patients with type 2 diabetes mellitus, dyslipidemia and periodontitis
Source: Sci Rep. 2020 May 18;10:8145. doi: 10.1038/s41598-020-65042-9 (PMC7235087; doi:10.1038/s41598-020-65042-9)
Supplement: Supplementary file 1 — Suplementary Information. [file 41598_2020_65042_MOESM1_ESM.docx]

**Supplementary Material**

**Circulating lymphocytes and monocytes transcriptomic analysis of patients with type 2 diabetes mellitus, dyslipidemia and periodontitis**

Sâmia C. T. Corbi^1,2^, Jaira F. de Vasconcellos^3,#^ , Alliny S. Bastos^1^, Diego G. Bussaneli^2^, Bárbara Roque da Silva^2^, Raquel A. Dos Santos^4^, Catarina S. Takahashi^5,6^, Cristiane de S. Rocha^7^, Benilton de Sá Carvalho^8^, Cláudia V. Maurer-Morelli^7^, Silvana R. P. Orrico^1^, Silvana P. Barros^9^, Raquel M. Scarel-Caminaga^2*^

**Supplementary Materials and Methods**

***Isolation of peripheral blood mononuclear cells and RNA extraction***

Peripheral venous blood collected from each subject was immediately submitted to centrifugation on a Ficoll-Paque PLUS (GE Healthcare Life Sciences, Oslo, Norway) density gradient and consecutive washing with saline (NaCl 0.9%) to isolate peripheral blood mononuclear cells (PBMCs). Total RNA was extracted using the Trizol® reagent (Invitrogen, Rockville, MD, USA) according to manufacturer's instructions. Samples containing total RNA were purified by RNeasy Protection Mini Kit (Qiagen, Hilden, Germany). RNA was quantified by NanoVue Spectrophotometer (GE Healthcare Life Sciences, Oslo, Norway) and its integrity was assessed by agarose gel electrophoresis (1%). Only RNA samples in the λ(260/280) and λ(260/230) reasons between 1.8 to 2.2 were used for microarray and quantitative real time PCR analyses. RNA samples were stored at -80° C until further downstream analyses ^1^.

***Oligonucleotide Microarray Analysis and Gene Enrichment Analyses***

Microarray data was generated from patients of T2DMpoorly-DL-P (n=5), T2DMwell-DL-P (n=7), DL-P (n=6), P (n=6) and H (n=6), after considering greater homogeneity regarding biochemical, lipid and clinical periodontal parameters ^2^ Five hundred nanograms of RNA were used as initial input for each sample in GeneChip IVT Labeling Kit protocol and hybridized to U133 Plus 2.0 (Affymetrix Inc., Santa Clara, CA, USA) arrays, which comprises 54,675 human transcripts. The U133 Plus 2.0 arrays were scanned twice using the GeneChip Scanner 3000 7G (Affymetrix Inc., Santa Clara, CA, USA) ^1^.

The raw CEL files were processed using the RMA method, as implemented in the Affy ^3^ package. The workflow performs background correction, quantile normalization and summarization via median polish. The final result is a set of quantities that are proportional to the probeset loT2DMwell-DL-P-expression (Irizarry et al. 2003). These log2-expression quantities resulted from the Robust Multi-array Average (RMA) method were further processed by the RankProd package ^4^. This methodology allows one to identify genes that show evidences for up- and downregulation with respect to a reference group ^4^ . To quantify the evidences showed by the data, we used a non-parametric approach based on 1,000 permutations to estimate the percentage of false prediction (pfp) of differential expression. Probesets that presented log_2⁡FC>1 and pfp<0.01 were called up- or downregulated, depending on the sign of the log-FC. Pairwise comparisons were made between the H (systemically and oral healthy subjects) and each of the T2DMpoorly-DL-P, T2DMwell-DL-P, DL-P and P groups (i.e. T2DMpoorly-DL-P versus H; T2DMwell-DL-P versus H; DL-P versus H and P versus H).

The probesets that were flagged on the aforementioned workflow were processed in a gene enrichment analysis through the Ingenuity Pathway Analysis software (IPA; Build 470319M, Version 43605602, Qiagen, Redwood City, CA). This strategy allows the identification of over-represented classes of genes that are based on their biological functional relevance.

Additionaly, the list of probes from the gene expression profiles of each patient group and control (H) were applied to GSEA software version 4.0.3 (http://www.broad.mit.edu/gsea). A .gmt file containing curated gene sets version 7.0 (c2.all.v7.0.symbols.gmt) was downloaded from MSigDB database. Curated gene sets were queried against each group gene expression profiles with the following parameters: Collapse/Remap to gene symbols = Collapse; Permutation type = gene_set; Number of permutations = 1000; Phenotype labels = control vs. each patient group; Enrichment statistic = weighted; Metric for ranking genes = Signal2Noise; Min. size = 15; Max. size = 500. Gene sets were considered significant enriched when p<0.05, FDR q-value<0.05 and FWER p-value<0.05. Details of the GSEA software and algorithms are provided elsewhere ^5^

From these microarray and enrichment analyses pairwise comparisons, to select candidate DEGs to an independent validation, it was considered the IPA report analysis, mainly the Top Analysis-Ready Molecules: expression fold change up or down regulated molecules. Then, from those molecules, three to six of candidate DEGs were chosen to an independent validation, based on their potential biological relation with one or more of the pathologies investigated here.

***Reverse transcription-quantitative polymerase chain reaction (RT-qPCR) Real-Time Analysis***

In order to make an independent validation of selected circulating lymphocytes and monocytes DEGs as possibly relevant after both microarray and functional enrichment analysis, we conducted RT-qPCR analyses. Reverse transcription reactions were made utilizing the SuperScript III First Strand Synthesis SuperMix (Invitrogen). Complementary DNA (cDNA) was used to perform qPCR reactions for the selected DEGs: TGB1I1 (Hs00210887_m1), VNN1 (Hs01546812_m1), CAVIN2 (HS00190538_m1), HLADRB4 (Hs03027795_uH), UGCG (Hs00916612_m1), CXCL8/IL8 (Hs00174103_m1), FN1 (Hs01549976_m1), BPTF (Hs00925866_m1), PDE3B (Hs00265322_m1), DAB2 (Hs01120074_m1), IGHDL-P (Hs00941519_g1), CD47 (Hs00179953_m1), and ITGB2 (Hs00164957_m1). As endogenous controls of the qPCR reactions, we identified three genes ACTG1 (Hs03044422_ g1), RPL7A (Hs00605223_ g1) and GAPDH (Hs02758991_ g1), which demonstrated best housekeeping expression pattern considering the five different studied groups ^2^.

All patients of each group (n=30 each group, including the patients chosen for microarray analysis) totalizing 150 patients were included in the microarray validation by RT-qPCR. All the reactions were performed in duplicate utilizing TaqMan® gene expression assays in the 7500 Real-Time PCR-System (Thermo Fisher Scientific, Foster City, CA, USA). To calculate gene expression, the Expression Suite Software was used (Applied Biosystems, Foster City, CA, USA), which employs the comparative Cτ (ΔCτ) method for multiplate data analysis ^2^.

***Statistical analysis***

The distribution and normality of the demographic and clinical variables were evaluated by the D’Agostino-Pearson test. The general characteristics of each group were described by mean and standard deviation (SD). The Chi-squared test was used to compare the gender distribution among the groups, and the Kruskall-Wallis test (followed by Dunn’s post-test) for the other characteristics ^1^. For the RT-qPCR analyses, values of 2-ΔCτ were compared between each two groups by the Mann-Whitney test using a significance level of 0.05. Statistical analyses were carried out in the GraphPad Prism software, version 5.0 ^2^.

**Supplementary Results**

The Supplementary Table 1 summarizes the top canonical pathways identified in circulating lymphocytes and monocytes of each pairwise group comparison. Noteworthy, results in Table 2 of the main text and the Supplementary Table 1 showed repeatedly the immunological and inflammatory content, which are in agreement with some of the enriched networks illustrated in the Figures 1 (A and B) and 2 (A and B) in the main text.

**Supplementary Table 1.** Top Canonical Pathways identified by Ingenuity Pathway Analysis (IPA) in circulating lymphocytes and monocytes of each pairwise comparison of the studies groups

| **Pairwise comparison** | **Name/Pathways** | ***p*-value** | **Overlap** |
| --- | --- | --- | --- |
| **T2DMpoorly-DL-P *versus* H** | Granulocyte Adhesion and Diapedesis | 8.10E-06 | 12.4% 20/161 |
|  | Pathogenesis of Multiple Sclerosis | 1.17E-05 | 55.6% 5/9 |
|  | Macropinocytosis Signaling | 2.06E-05 | 16.0% 13/81 |
|  | Inhibition of Angiogenesis by TSP1 | 3.05E-05 | 25.0% 8/32 |
|  | HER-2 Signaling in Breast Cancer | 5.07E-05 | 14.8% 13/88 |
| **T2DMwell-DL-P *versus* H** | B Cell Development | 88.99E-06 | 25.9% 7/27 |
|  | Communication between Innate and Adaptive Immune Cells | 1.50E-05 | 14.1% 11/78 |
|  | Airway Pathology in Chronic Obstructive Pulmonary Disease | 4.50E-05 | 50.0% 4/8 |
|  | Granulocyte Adhesion and Diapedesis | 7.30E-05 | 9.3% 15/161 |
|  | Allograft Rejection Signaling | 9.69E-05 | 18.4% 7/38 |
| **DL-P *versus* H** | Integrin Signaling | 4.01E-04 | 5.7% 12/211 |
|  | Allograft Rejection Signaling | 5.28E-04 | 13.2% 5/38 |
|  | Clathrin-mediated Endocytosis Signaling | 8.07E-04 | 5.6% 11/197 |
|  | Sertoli Cell-Sertoli Cell Junction Signaling | 8.89E-04 | 5.9% 10/169 |
|  | Cdc42 Signaling | 1.42E-03 | 6.6% 8/121 |
| **P *versus* H** | Communication between Innate and Adaptive Immune Cells | 2.04E-06 | 12.8% 10/78 |
|  | B Cell Development | 9.19E-06 | 22.2% 6/27 |
|  | Primary Immunodeficiency Signaling | 1.04E-05 | 17.1% 7/41 |
|  | Hematopoiesis from Pluripotent Stem Cells | 4.44E-05 | 17.1% 6/35 |
|  | Allograft Rejection Signaling | 7.20E-05 | 15.8% 6/38 |
|  |  |  |  |

**Supplementary Figure 1.** Hierarchical clustering of PBMC genes with significantly different expression changes between **(A)** T2DMpoorly-DL-P *versus* H, **(B)** T2DMwell-DL-P *versus* H, **(C)** DL-P *versus* H and **(D)** P *versus* H. Red and green indicate post-event up- and down-regulation, respectively. Intensity of color indicates the degree of gene regulation.


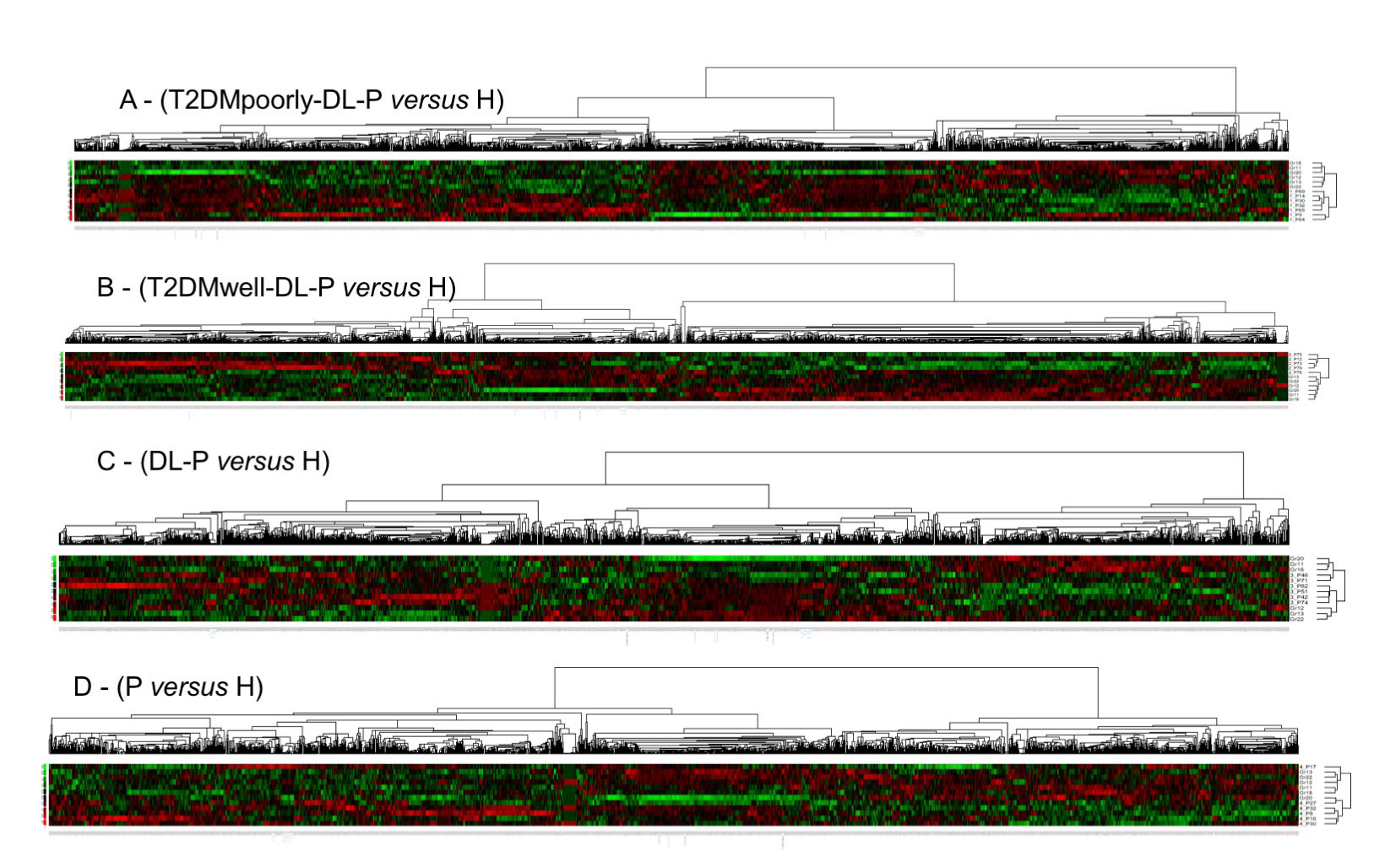


**Supplementary Discussion**

The validation by RT-qPCR of the PBMC expression profiling of T2DMpoorly-DL-P *versus* H, demonstrated that *VNN1* was significantly upregulated in T2DMpoorly-DL-P. The *VNN1* *(Vanin 1)* gene, a member of the Vanin family of proteins, participate in hematopoietic cell trafficking and may play a role in oxidative-stress response ^6^. Our present findings support a previously reported oxidative-stress response function for *VNN1*, since oxidative stress is intrinsically related to the pathogenesis of T2DM, dyslipidemia and periodontitis ^1,7^. In agreement, we previously showed higher irreversible damage to DNA molecule in T2DMpoorly-DL-P than in H subjects utilizing the mutagenesis approach (micronucleus test), suggesting that patients concurrently presenting these three pathologies (T2DMpoorly-DL-P) may have antioxidant defense deficiency compared to healthy individuals (H) ^8^. Considering that the T2DMpoorly-DL-P subjects present dyslipidemia, Goring et al. (2007) ^9^ reported that *VNN1* gene expression levels showed the strongest correlation with HDL-C (high-density lipoprotein cholesterol) ^10^ concentrations in lymphocytes of Mexican-American adults. In addition, there is experimental evidence that *VNN1* gene is a central regulator of lipid biosynthesis by controlling the flux through the fatty acid and/or cholesterol biosynthetic pathways ^10,11^.

The circulating lymphocytes and monocytes of T2DM patients with good glycemic control also affected by dyslipidemia and periodontitis (T2DMwell-DL-P) compared with systemic and oral healthy subjects demonstrated RT-qPCR validated results regarding the *BPTF* and *PDE3B* genes, which were upregulated in H, while the *FN1* gene was validated as upregulated in the T2DMwell-DL-P group (Figure 5B). The *BPTF (Bromodomain PHD Finger Transcription Factor)* gene encodes a protein with a zinc finger motif and a DNA-binding domain, which is suggested to play a role in cell proliferation during the regulation of transcription. The encoded protein is highly similar to transcription factors that recognize H3 tails trimethylated on Lys-4 (H3K4me3), which mark transcription start sites of virtually all active genes, binding directly to DNA^12^. Despite the fact that in the literature the *BPTF* gene has been associated with neurodegenerative diseases, previous studies correlating the PBMC expression of this gene with any of the three chronic inflammatory diseases studied here were not found. Further studies with larger and more diverse ethnic populations should be performed to confirm and strengthen our findings. Moreover, the *PDE3B (Phosphodiesterase 3B)* gene, which was downregulated in T2DMwell-DL-P compared to H subjects, is known to be involved in signaling and regulation of lipolysis in adipocytes, whereupon this gene may play a role in fat metabolism^13^. In addition, it seems that the reduction of the *PDE3B* gene expression could lead to insulin resistance through elevated serum free fatty acids (FFA) ^14^. This is in agreement with our validated findings of the *PDE3B* gene downregulation in the circulating lymphocytes and monocytes of T2DMwell-DL-P group. Furthermore, the functional enrichment analysis by IPA comparing T2DMwell-DL-P *versus* H subjects, showed the *PDE3B* gene (downregulated in T2DMwell-DL-P) on the same important network (Figure 1B) of cell death and survival, organismal injury and abnormalities, inflammatory response, together with the *FN1* gene (upregulated in T2DMwell-DL-P subjects). The *FN1 (Fibronectin 1)* gene, validated as upregulated in PBMC of T2DMwell-DL-P group, encodes a glycoprotein named fibronectin, which is present in plasma in a soluble dimeric form, while at the cell surface and in extracellular matrix the fibronectin is present in a dimeric or multimeric form. Fibronectin is involved in cell adhesion and migration processes including wound healing, blood coagulation, host defense, and metastasis ^15^. Concerning mainly the wound healing and host defense, our present circulating lymphocytes and monocytes expression results suggest that the *FN1* overexpression in T2DMwell-DL-P subjects could indicate a more overstated immune/inflammatory response than in health individuals, as well as an attempt to compensate for wound healing, that is recognized to be delayed. Also, diseases associated with *FN1* gene include glomerulopathy with fibronectin deposits 2, including diabetic nephropathy ^16,17^.

In the DL-P *versus* H comparison, which evaluated the effect of dyslipidemia associated with periodontitis, the validated genes expressing in PBMC were *CD47* and *DAB 2*. The *DAB2* (Clathrin Adaptor Protein) encodes a mitogen-responsive phosphoprotein playing role in cellular differentiation, in endocytosis of integrin beta-1, besides a potential tumor suppressor ^18^. In the modulation of integrins the *CD47 (CD47 Molecule)* gene validated in the DL-P is also involved in increased intracellular calcium concentration that occurs upon cell adhesion to the extracellular matrix. Among the *CD47* related pathways are the innate immune system and integrins modulation, but no information was found in the literature regarding PBMC expression of *CD47* as well as *DAB2* and their potential role in the pathogenesis of T2DM, dyslipidemia and periodontitis.

Lastly, the comparison of P *versus* H, which evaluated the influence of the presence of periodontitis, demonstrated the validation of the *ITGB2 (Integrin Subunit Beta 2)* and the *Immunoglobulin Heavy Constant Gamma 3 (IGHDL-P)* genes (Figure 6B) in circulating lymphocytes and monocytes of patients. The *ITGB2 (Integrin Subunit Beta 2)* gene encodes a beta chain of the integrin protein, which forms different integrin heterodimers after to be combined with multiple alpha chains. Integrins are integral cell-surface proteins that participate in cell adhesion as well as cell-surface mediated signaling. The encoded protein plays an important role in immune response, and integrin alpha-L/beta-2 is a receptor for ICAM-1, -2, -3 and -4^19^. The comparison of P *versus* H, which evaluated the influence of the presence of periodontitis, demonstrated the validation of the *IGHDL-P* gene upregulation in the P group (Figure 6B). The *IGHDL-P (Immunoglobulin Heavy Constant Gamma 3)* gene is related to pathways like role of phospholipids in phagocytosis and innate immune system, since this gene encodes a constant region of immunoglobulin G heavy chain ^20^. The IgG role in periodontitis is known mainly regarding the IgG antibodies to *Porphyromonas gingivalis* (*P.g*) or *Aggregatibacter actinomycetemcomitans (A.a.).* Systemic levels of these IgG antibodies were associated with cardiovascular disease and periodontitis in univariate analyses, but only the association of *P.g.* IgG antibody levels with periodontitis reached statistical significance after adjustment for common confounders ^21,22^. However, we were not able to state that high *IGHDL-P* mRNA levels in the PBMC of our periodontitis (P) patients group occurred because of *P.g.* and/ or *A.a.* infection, even though elevated levels of circulating antibodies against these periodontopathogens in patients with periodontitis have consistently been reported ^22-27^, as reviewed by Damgaard et al. (2017) ^21^.

**Supplementary References**

1 Corbi, S. C. *Avaliação da expressão gênica e de lesões no DNA de índividuos portadores de diabetes mellitus tipo 2, dislipidemia e periodontite crônica* PhD thesis, Universidade Estadual Paulista Julio de Mesquita Filho, Faculdade de Odontologia de Araraquara, (2014).

2 Corbi, S. C. T. *et al.* Expression Profile of Genes Potentially Associated with Adequate Glycemic Control in Patients with Type 2 Diabetes Mellitus. *J Diabetes Res* **2017**, 2180819, doi:10.1155/2017/2180819 (2017).

3 Gautier, L., Cope, L., Bolstad, B. M. & Irizarry, R. A. affy--analysis of Affymetrix GeneChip data at the probe level. *Bioinformatics* **20**, 307-315, doi:10.1093/bioinformatics/btg405 (2004).

4 Breitling, R., Armengaud, P., Amtmann, A. & Herzyk, P. Rank products: a simple, yet powerful, new method to detect differentially regulated genes in replicated microarray experiments. *FEBS Lett* **573**, 83-92, doi:10.1016/j.febslet.2004.07.055 (2004).

5 Subramanian, A. *et al.* Gene set enrichment analysis: a knowledge-based approach for interpreting genome-wide expression profiles. *Proc Natl Acad Sci U S A* **102**, 15545-15550, doi:10.1073/pnas.0506580102 (2005).

6 *GeneCards – the human gene database VNN1*, <<http://www.genecards.org/cgi-bin/carddisp.pl?gene=VNN1&keywords=VNN1>.> (2019).

7 Soory, M. Inflammatory mechanisms and redox status in periodontal and cardiometabolic diseases: effects of adjunctive nutritional antioxidants and statins. *Infect Disord Drug Targets* **12**, 301-315 (2012).

8 Corbi, S. C. *et al.* Elevated micronucleus frequency in patients with type 2 diabetes, dyslipidemia and periodontitis. *Mutagenesis* **29**, 433-439, doi:10.1093/mutage/geu043 (2014).

9 Goring, H. H. *et al.* Discovery of expression QTLs using large-scale transcriptional profiling in human lymphocytes. *Nat Genet* **39**, 1208-1216, doi:10.1038/ng2119 (2007).

10 Jacobo-Albavera, L. *et al.* VNN1 gene expression levels and the G-137T polymorphism are associated with HDL-C levels in Mexican prepubertal children. *PLoS One* **7**, e49818, doi:10.1371/journal.pone.0049818 (2012).

11 Kaskow, B. J., Proffitt, J. M., Blangero, J., Moses, E. K. & Abraham, L. J. Diverse biological activities of the vascular non-inflammatory molecules - the Vanin pantetheinases. *Biochem Biophys Res Commun* **417**, 653-658, doi:10.1016/j.bbrc.2011.11.099 (2012).

12 *GeneCards – the human gene database BPTF*, <<http://www.genecards.org/cgi-bin/carddisp.pl?gene=BPTF&keywords=BPTF>.> (2019).

13 *GeneCards – the human gene database PDE3B*, <<http://www.genecards.org/cgi-bin/carddisp.pl?gene=PDE3B&keywords=PDE3B>.> (2019).

14 Osawa, H. *et al.* Systematic search for single nucleotide polymorphisms in the 5' flanking region of the human phosphodiesterase 3B gene: absence of evidence for major effects of identified polymorphisms on susceptibility to Japanese type 2 diabetes. *Mol Genet Metab* **79**, 43-51 (2003).

15 *GeneCards – the human gene database FN1*, <<http://www.genecards.org/cgi-bin/carddisp.pl?gene=FN1&keywords=FN1>.> (2019).

16 Huang, X. *et al.* Suppression of mesangial cell proliferation and extracellular matrix production in streptozotocin-induced diabetic mice by adiponectin in vitro and in vivo. *Horm Metab Res* **46**, 736-743, doi:10.1055/s-0034-1375626 (2014).

17 Lv, C. *et al.* The changes in miR-130b levels in human serum and the correlation with the severity of diabetic nephropathy. *Diabetes Metab Res Rev* **31**, 717-724, doi:10.1002/dmrr.2659 (2015).

18 *GeneCards – the human gene database DAB2*, <<http://www.genecards.org/cgi-bin/carddisp.pl?gene=DAB2&keywords=DAB2>.> (2019).

19 *GeneCards – the human gene database ITGB2*, <<http://www.genecards.org/cgi-bin/carddisp.pl?gene=ITGB2&keywords=ITGB2>.> (2019).

20 *GeneCards – the human gene database IGHG3*, <<http://www.genecards.org/cgi-bin/carddisp.pl?gene=IGHG3&keywords=IGHG3>.> (2019).

21 Damgaard, C. *et al.* Immunoglobulin G antibodies against Porphyromonas gingivalis or Aggregatibacter actinomycetemcomitans in cardiovascular disease and periodontitis. *J Oral Microbiol* **9**, 1374154, doi:10.1080/20002297.2017.1374154 (2017).

22 Chung, H. Y. *et al.* Immunoglobulin G profiles in different forms of periodontitis. *J Periodontal Res* **38**, 471-476 (2003).

23 Dye, B. A. *et al.* Serum antibodies to periodontal bacteria as diagnostic markers of periodontitis. *J Periodontol* **80**, 634-647, doi:10.1902/jop.2009.080474 (2009).

24 Graswinckel, J. E., van der Velden, U., van Winkelhoff, A. J., Hoek, F. J. & Loos, B. G. Plasma antibody levels in periodontitis patients and controls. *J Clin Periodontol* **31**, 562-568, doi:10.1111/j.1600-051X.2004.00522.x (2004).

25 Hyvarinen, K. *et al.* Detection and quantification of five major periodontal pathogens by single copy gene-based real-time PCR. *Innate Immun* **15**, 195-204, doi:10.1177/1753425908101920 (2009).

26 Pussinen, P. J., Vilkuna-Rautiainen, T., Alfthan, G., Mattila, K. & Asikainen, S. Multiserotype enzyme-linked immunosorbent assay as a diagnostic aid for periodontitis in large-scale studies. *J Clin Microbiol* **40**, 512-518 (2002).

27 Wang, D. *et al.* Elevated serum IgG titer and avidity to Actinobacillus actinomycetemcomitans serotype c in Japanese periodontitis patients. *Oral Microbiol Immunol* **20**, 172-179, doi:10.1111/j.1399-302X.2005.00208.x (2005).
